# Multiplex Single-Nucleotide Microbial Genome Editing Achieved by CRISPR-Cas9 Using 5′-End-Truncated sgRNAs

**DOI:** 10.1021/acssynbio.3c00323

**Published:** 2023-06-27

**Authors:** Se Ra Lim, Ho Joung Lee, Hyun Ju Kim, Sang Jun Lee

**Affiliations:** Department of Systems Biotechnology and Institute of Microbiomics, Chung-Ang University, Anseong 17546, Republic of Korea

**Keywords:** CRISPR-Cas, multiplex, single-nucleotide editing, truncated sgRNA

## Abstract

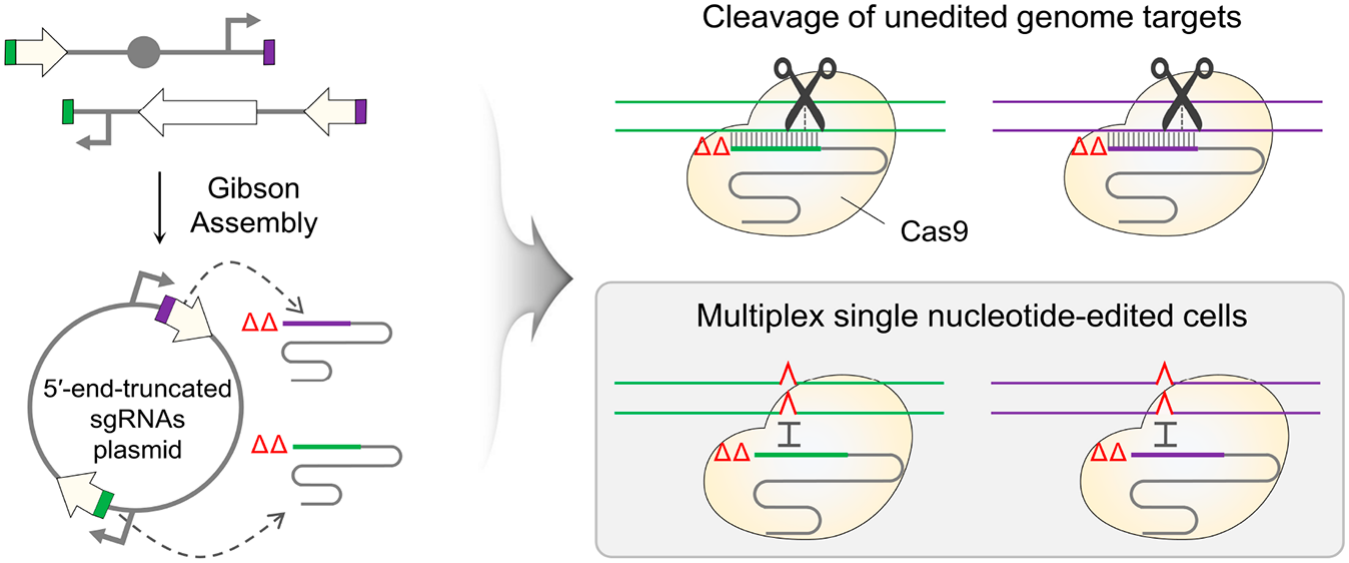

Multiplex genome editing with CRISPR-Cas9 offers a cost-effective solution for time and labor savings. However, achieving high accuracy remains a challenge. In an *Escherichia coli* model system, we achieved highly efficient single-nucleotide level simultaneous editing of the *galK* and *xylB* genes using the 5′-end-truncated single-molecular guide RNA (sgRNA) method. Furthermore, we successfully demonstrated the simultaneous editing of three genes (*galK*, *xylB*, and *srlD*) at single-nucleotide resolution. To showcase practical application, we targeted the *cI*^*857*^ and *ilvG* genes in the genome of *E. coli*. While untruncated sgRNAs failed to produce any edited cells, the use of truncated sgRNAs allowed us to achieve simultaneous and accurate editing of these two genes with an efficiency of 30%. This enabled the edited cells to retain their lysogenic state at 42 °C and effectively alleviated l-valine toxicity. These results suggest that our truncated sgRNA method holds significant potential for widespread and practical use in synthetic biology.

## Introduction

The CRISPR-Cas system consists of a guide RNA (gRNA) module that recognizes a target sequence and a Cas nuclease that cuts phosphodiester bonds in the target nucleic acids and has been developed as a useful genome editing tool from bacteria through humans for the past decade.^1^ To save time and labor in the iterative process for each gene editing, multiplex genome editing could be achieved by the expression of multiple gRNAs. It is also possible to reduce the probability of unwanted mutations accumulating in cells by avoiding the repetition of gene inactivation associated with the DNA repair system or the overexpression of recombinase for more efficient editing.

Multiplex microbial genome editing using CRISPR-Cas technology was first reported in *Streptococcus pneumoniae*, and two target genes were deleted with 75% efficiency.^2^ Since then, multiplex genome editing technology in microorganisms has been widely used. However, it is very rare to encounter cases where single-nucleotide point mutations were introduced in several genes simultaneously.^3^ Highly efficient single-nucleotide-level editing in microbial cells was recently accomplished by truncated gRNA methods, which are relatively practical and straightforward methods to implement.^4−6^ In this study, we report CRISPR-Cas9-mediated multiplex single-nucleotide-level genome editing in the genome of *Escherichia coli* using 5′-end-truncated single-molecular guide RNAs (sgRNAs).

## Results and Discussion

### CRISPR-Cas9-Mediated Cleavage of Multiple Targets in the Microbial Genome

Site-specific mutagenesis was induced simultaneously in the *galK* and *xylB* genes of the *E. coli* genome using single-strand oligonucleotides (Figure S1A, Supporting Information). If mutagenesis occurred simultaneously in the *galK* and *xylB* genes, the sgRNA/Cas9 complex could not recognize the edited sequence as a target, and the cell survives. When a mutation is introduced into only one gene or when mutations are not introduced into both genes, the cell is eliminated by sgRNA/Cas9 (Figure S1B). Because the mutations were designed to induce stop codons in the *galK* and *xylB* genes, surviving cells with the desired mutations could not use d-galactose and d-xylose, forming white colonies on MacConkey agar containing the two carbon sources.

We designed a multiplex sgRNA plasmid to express two sgRNAs targeting the *galK* and *xylB* genes, respectively (Figure S2A). In most cases, gRNA cassettes are arranged and expressed in tandem for multiplex genome editing, but simultaneous editing may fail because some of the gRNA sequences are lost due to intra- or intermolecular recombination.^7,8^ To address this issue, in our study, each sgRNA cassette was placed between the *ori* region and the antibiotic resistance marker gene to allow the replicating sgRNA plasmid to maintain the sgRNA sequences under antibiotic conditions. We verified whether each sgRNA expressed by the dual sgRNA plasmid can independently form an active complex with Cas9 nuclease to recognize and cleave each target (Figure S2B).

Simultaneous 1–4 nt substitutions were performed on the *galK* and *xylB* genes. In our previous studies, we used a mutagenic oligonucleotide that was 41 nucleotides long and was used at an amount of 100 pmol, which allowed for highly efficient editing.^4−6^ However, despite these conditions, we were unable to obtain cells with coedited mutations. When the oligonucleotide length was increased to 70-mer and the amount was increased to 500 pmol, we observed that the white colony ratio increased (Figure S3). In the case of a 4 nt substitution, 74% of white colonies were confirmed under the 70-mer, 500 pmol condition. However, the mutagenesis efficiency did not continuously increase in proportion to the oligonucleotide length (Figure S4). This is probably because the longer the oligonucleotide, the lower the cellular delivery efficiency. Even when the mutagenesis efficiency was optimized by adjusting the length and amount of oligonucleotide, white colonies with 1 nt substitution in each of the *galK* and *xylB* genes could not be observed. This result is probably due to the failure of negative selection by recognizing both the single-nucleotide-edited target and the unedited target due to the mismatch tolerance of CRISPR-Cas9.^4^ In addition, when the white colonies obtained from the 2 or 3 nt substitution experiment were confirmed by Sanger sequencing, indels were observed in the target sequence, besides the desired mutation in 2 out of 4 colonies (Figure S5).

### Multiplex Single-Nucleotide Editing Using 5′-End-Truncated sgRNAs

**Figure 1:**
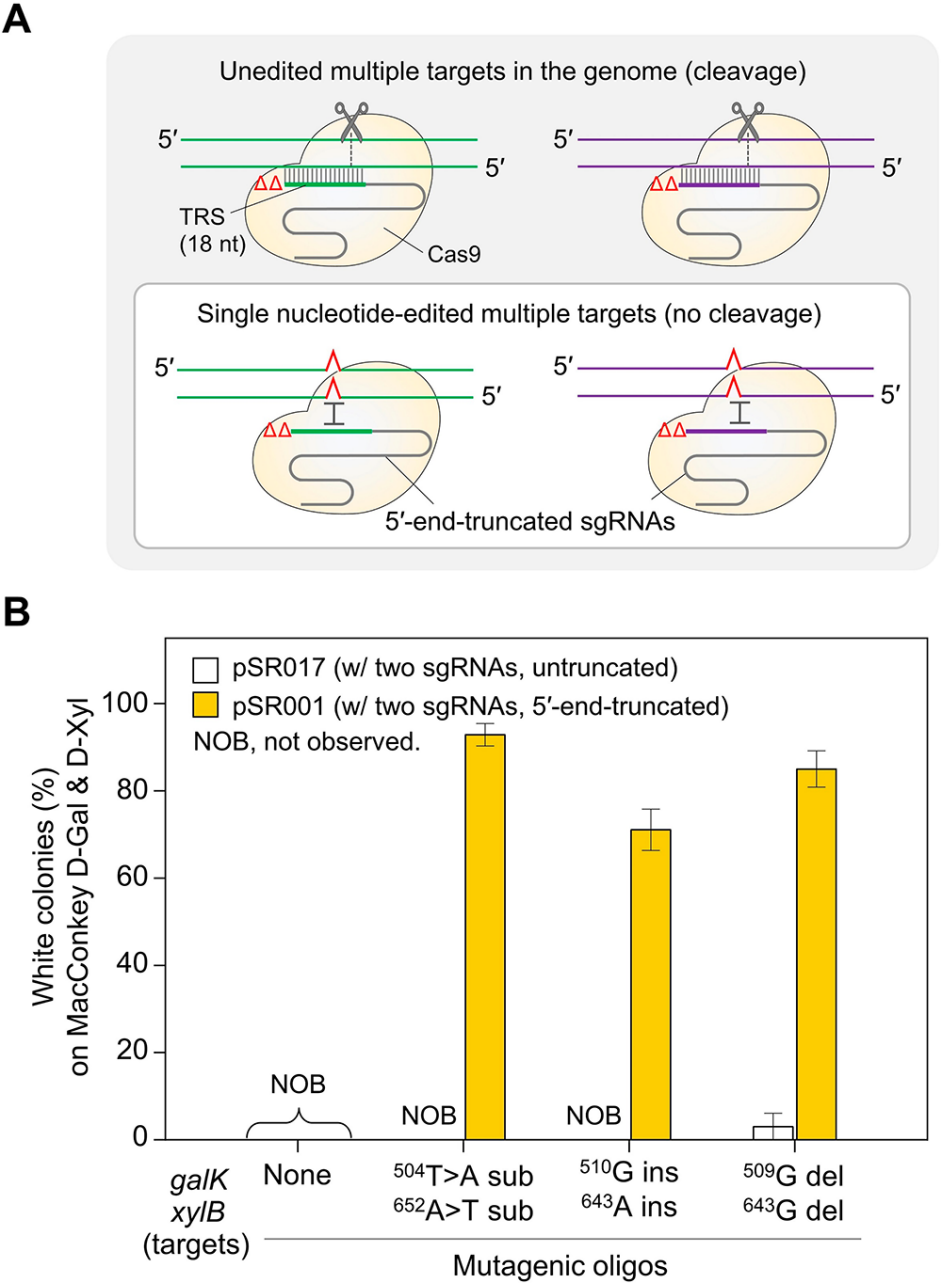
Multiplex single-nucleotide editing in *galK* and *xylB* using 5′-end-truncated sgRNAs. (A) Cas9-mediated negative selection of single-nucleotide-edited multiple targets using 5′-end-truncated sgRNAs. The 5′-end-truncated sgRNA/Cas9 complex recognizes and cleaves unedited targets. Single-nucleotide-edited targets are not cleaved by the 5′-end-truncated sgRNA/Cas9 complex. (B) Editing efficiencies of multiplex single-nucleotide editing in the *galK* and *xylB* genes using untruncated or 5′-end-truncated sgRNAs. The types of edits observed included substitutions (sub), insertions (ins), and deletions (del).

To achieve single-nucleotide editing at both *galK* and *xylB* genes, we harnessed the 5′-end-truncated sgRNA method. Because the 5′-end-truncated sgRNA/Cas9 complex does not cause cleavage if there is only one mismatch in the target DNA, it was expected that cells in which single-nucleotide editing was successful in both genes at the same time could be negatively selected (Figure 1A). A multiplex sgRNA plasmid carrying 5′-end-truncated sgRNAs was constructed, and the cleavage of the genome by Cas9 associated with the dual sgRNA plasmid was also confirmed (Figure S6). The number of surviving cells was slightly higher than those of Δ*galK* or Δ*xylB* cells when untruncated sgRNAs were used (Figure S2B). It is thought that this may be due to the reduced *in vivo* activity of the 5′-end-truncated sgRNA/Cas9 complex compared to that of the original sgRNA/Cas9 complex.^9^

When untruncated sgRNAs were employed, the presence of white colonies, indicating single-nucleotide edits, was rarely observed (0–3%) (Figure 1B). However, when 5′-end-truncated sgRNAs were used, the frequencies of single-nucleotide substitutions, insertions, and deletions were found to be 93, 71, and 85%, respectively. These values were determined based on the count of white colonies observed on MacConkey agar containing d-galactose and d-xylose. As a result of Sanger sequencing, only the desired mutation was introduced in all identified white colonies (Figure S7).

Additionally, we performed simultaneous single-nucleotide editing on three targets. A plasmid expressing three sgRNAs targeting *galK*, *xylB*, and *srlD* was designed to prevent the loss of three sgRNA sequences (Figure S8A). For the *srlD* target gene, we tested whether single-nucleotide editing at the *srlD* gene is possible using 5′-end-truncated sgRNA. As a result, on the MacConkey medium containing d-sorbitol, the white colony ratio was 8 and 68%, respectively, and edited DNA sequences were confirmed (Figure S9). When using a plasmid carrying three untruncated sgRNAs, white colonies indicating single- or quadruple-nucleotide-substituted cells could not be obtained on MacConkey agar containing d-galactose, d-xylose, and d-sorbitol (Figure S8B). When employing 5′-end-truncated sgRNAs for each of the three targets, the percentage of cells with simultaneous 1 and 4 nt substitutions were 9 and 13%, respectively. Notably, the ratio of white colonies showing single-nucleotide substitutions was significantly reduced compared to when only *galK* and *xylB* genes were edited (93%) (Figure 1B). The lack of significant differences in the efficiencies of 1 and 4 nt substitutions suggests that neither the operation of the sgRNA/Cas9 complex nor negative selection is a problem. In general, as the number of targets increases, the editing efficiency decreases.^3^ The low probability of negative selection after the completion of each mutagenesis at the three gene loci may explain why the simultaneous editing efficiency of three genes is not as high as that of two genes. Although the editing efficiency was not high, accurate single-nucleotide editing at three different loci was confirmed in randomly selected white colonies using Sanger sequencing (Figure S8C).

### Simultaneous Restoration of *cI*^*857*^ and *ilvG*^–^ Mutations Using the 5′-End-Truncated sgRNA Method

To assess the applicability of this accurate multiplex genome editing method in real world scenarios, the *cI*^*857*^ gene (encoding a temperature-sensitive lysogenic switch) and *ilvG* gene (involved in l-valine metabolism) were selected as targets. Candidate colonies that underwent multiplex editing were randomly selected from an LB agar containing spectinomycin (75 μg/mL). The *cI*^*857*^ mutation confers the ability for λ lysogenic cells to enter the lytic cycle at 42 °C. If the *cI*^*857*^ mutation is reversed to wild type through single-nucleotide substitution (^199^A to G), λ lysogenic *cI*^*WT*^ cells remained lysogenic at both 30 and 42 °C. A frameshift mutation in the *ilvG* gene leads to l-valine toxicity. By converting *ilvG*^–^ back to its wild-type form through a two-nucleotide insertion (^979^AT), the l-valine toxicity is alleviated.

**Figure 2:**
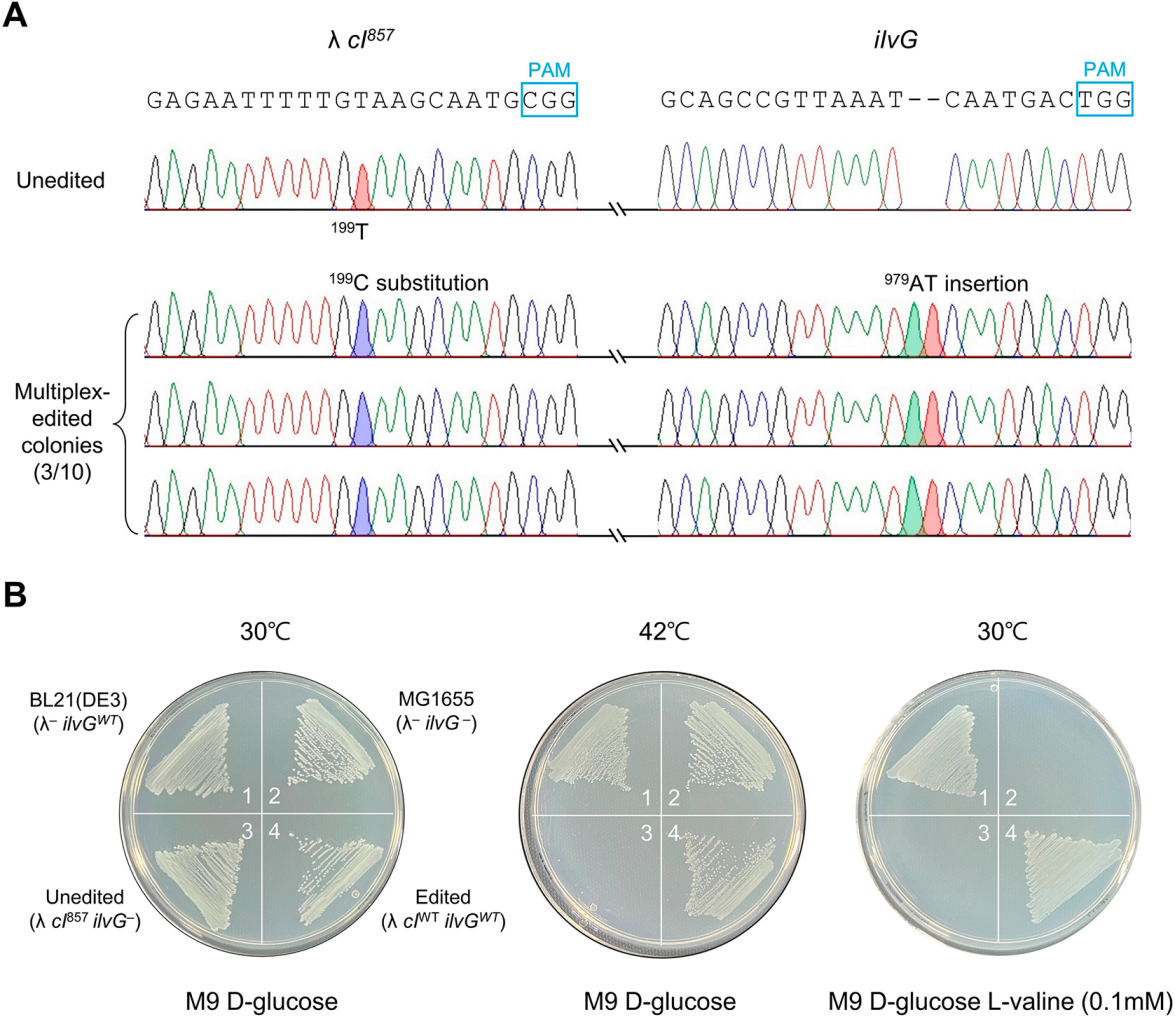
5′-End-truncated sgRNA/Cas9-mediated multiplex genome editing of *cI*^*857*^ and *ilvG*. (A) Unedited and edited DNA sequences of *cI*^*857*^ and *ilvG*. Editing target nucleotides are shaded on the chromatograms. (B) Phenotypic changes of multiplex-edited cells confirmed by the addition of l-valine (0.1 mM) in an M9 glucose medium or incubation at 42 °C. λ *cI*^*857*^ lysogenic cells undergo lysis at 42 °C, and *ilvG*^–^ cells are unable to grow when l-valine is added. 1, BL21(DE3) (λ^–^*ilvG*^*WT*^); 2, MG1655 (λ^–^*ilvG*^–^); 3, HL012 (MG1655, λ *cI*^*857*^*ilvG*^–^, unedited); 4, SR019 (MG1655, λ *cI*^*WT*^*ilvG*^*WT*^, multiplex-edited).

When using untruncated sgRNAs, multiplex-edited colonies could not be obtained among 10 randomly selected colonies. This was confirmed in an M9 glucose medium supplemented with l-valine (0.1 mM) or at an incubation temperature of 42 °C (Figure S10). However, when employing 5′-end-truncated sgRNAs, simultaneous editing of the *cI* and *ilvG* genes was achieved with an efficiency of 30%. Sanger sequencing of 10 randomly selected colonies revealed that 9 of them exhibited accurate editing of the *ilvG* gene without any undesired mutations, and the *cI* gene was correctly edited in 3 out of the same 10 colonies (Figure 2A). The phenotypic changes of randomly selected colonies were confirmed by the addition of l-valine (0.1 mM) in the M9 glucose medium or at an incubation temperature of 42 °C (Figure S11). Notably, the multiplex-edited cells (*cI*^*WT*^ and *ilvG*^*WT*^) displayed no lysis when subjected to a temperature of 42 °C and exhibited no l-valine toxicity (Figure 2B).

Several studies have reported the successful simultaneous deletion of several dozen nucleotides to 1 kb at two or more sites in the *E. coli* genome, demonstrating the feasibility of multiplex editing methods.^10−12^ Furthermore, high-efficiency multiplex gene interruption has been achieved, with reported efficiencies of up to 95% for two target genes and 19% for three target genes.^13^ Additionally, simultaneous editing of a few nucleotides across two or three targets has also been demonstrated.^14−17^ However, achieving multiplex genome editing at the single-nucleotide level has proven challenging without gRNA modification, primarily due to the mismatch tolerance of the CRISPR-Cas system. Nonetheless, with the advent of the 5′-end-truncated sgRNA method, the obstacles in performing single-nucleotide multiplex genome editing in *E. coli* are almost completely overcome.

## Materials and Methods

The materials and methods employed in this study are thoroughly described in the Supporting Information.

## Supporting Information Available

Materials and Methods; scheme, optimization process, editing efficiencies, and Sanger sequences of multiplex genome editing (Figures S1–S11); lists of strains, plasmids, primers, and mutagenic oligonucleotides used in this study (Tables S1–S4) (PDF)

## Abbreviations

Cas9: clustered regularly interspaced short palindromic repeats-associated protein 9
CRISPR: clustered regularly interspaced short palindromic repeats
sgRNA: single-molecular guide RNA
