# Supplementary material for: Multiplex Single-Nucleotide Microbial Genome Editing Achieved by CRISPR-Cas9 Using 5′-End-Truncated sgRNAs
Source: ACS Synth Biol. 2023 Jun 27;12(7):2203–7. doi: 10.1021/acssynbio.3c00323 (PMC10368013; doi:10.1021/acssynbio.3c00323)
Supplement: Supplementary file 1 — sb3c00323_si_001.pdf [file sb3c00323_si_001.pdf]

Supporting Information

Multiplex Single-Nucleotide Microbial Genome Editing Achieved by CRISPR-Cas9 Using 5′-End-Truncated sgRNAs

Se Ra Lim†, Ho Joung Lee†, Hyun Ju Kim and Sang Jun Lee*

Department of Systems Biotechnology and Institute of Microbiomics, Chung-Ang University, Anseong 17546, Republic of Korea

† These authors contributed equally to this work.

* Corresponding author

Table of contents

Materials and Methods **-S3**

Supplementary Figure S1. Scheme of oligonucleotide-directed multiplex genome editing by CRISPR-Cas9 mediated negative selection. **-S5**

Supplementary Figure S2. Two-target multiplex genome editing in *galK* and *xylB* using dual untruncated sgRNA plasmid. **-S6**

Supplementary Figure S3. Optimization of length and amount of oligonucleotides. **-S7**

Supplementary Figure S4. Editing efficiency depending on the length of oligonucleotides. **-S8**

Supplementary Figure S5. Chromatograms of *galK* and *xylB* in double- and triple-nucleotide substituted cells obtained using two untruncated sgRNAs. **-S9**

Supplementary Figure S6. Confirmation of surviving colonies by negative selection of dual 5′-end-truncated sgRNA plasmid targeting *galK* and *xylB*. **-S10**

Supplementary Figure S7. Chromatograms of *galK* and *xylB* in single-nucleotide substituted-, inserted-, and deleted-cells obtained using 5′-end-truncated sgRNAs. **-S11**

Supplementary Figure S8. Three-target multiplex single-nucleotide editing in *galK*, *xylB*, and *srlD* using 5′-end-truncated sgRNAs. **-S12**

Supplementary Figure S9. Single-nucleotide editing in *srlD* target. **-S13**

Supplementary Figure S10. Phenotypes of 10 randomly selected colonies after multiplex editing of *cI* and *ilvG* genes using untruncated sgRNAs. **-S14**

Supplementary Figure S11. Phenotypes of 10 randomly selected colonies after multiplex editing of *cI* and *ilvG* genes using 5′-end-truncated sgRNAs. **-S15**

Supplementary Table S1. Strains used in this study. **-S16**

Supplementary Table S2. Plasmids used in this study. **-S17**

Supplementary Table S3. Primers used in this study. **-S18**

Supplementary Table S4. Mutagenic oligonucleotides used in this study. **-S20**

References **-S22**

MATERIALS AND METHODS

Strains and Culture Conditions.

The *E. coli* strains used in this study are listed in Table S1. *E. coli* was cultured in LB broth (LPS solution, Cat. No. LB-05, Korea) or M9 minimal media (M9 Salts (3 g/L KH_2PO_4, 0.5 g/L NaCl, 6.8 g/L Na_2HPO_4, and 1 g/L NH_4Cl, BD Difco, Cat. No. 248510, USA), 1 mM $MgSO_4$, 0.5 mM $CaCl_2$) with D-glucose (0.4%, CAS No. 50-99-7) as a sole carbon source. Depending on the *ori* in the plasmids, each strain was cultured at 30 or 37 °C. HL012[1] derivatives were generally cultured at 30 °C, and specifically cultured at 42 °C to confirm the reverted *cI* phenotype. When confirming the *ilvG* gene editing, 0.1mM L-valine (CAS No. 72-18-4) was added. *E. coli* DH5α was used as a cloning host to construct sgRNA plasmids. *E. coli* K-12 BW25113 gene knockout mutants from the Keio collection[2] were bought through Open Biosystems (Lafayette, CO, USA). As needed, the medium was supplemented with 50 μg/mL of ampicillin, 12.5 μg/mL of chloramphenicol, 25 μg/mL of kanamycin, and 50 or 75 μg/mL of spectinomycin.

Plasmid Construction.

The plasmids used in experiments related to multiplex genome editing are listed in Table S2. Primers used for plasmid construction are listed in Table S3. Single sgRNA plasmid was constructed in the same way as described in our previous study.[3] pHK470 was created by inserting the sgRNA cassette downstream of the spectinomycin resistance gene of pTargetF (provided generously by Sheng Yang; Addgene plasmid #62226). pSR020 was constructed by replacing the spectinomycin resistance gene of pSR001 with the chloramphenicol resistance gene amplified from pACYC184.[4] Triple sgRNA plasmid was generated using pHK470 and pSR020 as templates and primers with target recognition sequence overhang. For effective ligation of the three fragments, the incubation time was extended to 8 hours while recovering after heat shock transformation. All plasmids were constructed with Gibson Assembly Master Mix (NEB, Cat. No. E2611, MA, USA), and the sequence of the sgRNA cassette was confirmed through Sanger sequencing.

Construction of Target-Knockout Strains.

To construct a Cas9-harboring strain in which a single target gene (*galK* or *xylB*) was knocked out, the P1*vir* phage (provided generously by Sankar Adhya at the NIH) was used to introduce mutations to HL026 via standard P1 transduction. The P1 lysates of JW0740 and JW3536 from the Keio collection[2] were used. To knock out both *galK* and *xylB*, the kanamycin resistance gene was removed from SR005 using pCP20,[5] and the P1 lysate of JW0740 was used to transduce SR008 as a recipient strain. After P1 transduction, colony PCR was performed to verify whether each target

gene was substituted with the kanamycin resistance (Km^R) cassette. The identified colony was purified three times on LB agar plates containing kanamycin and used in the experiment. Primers used for colony PCR are listed in Table S3.

Oligonucleotide-Directed Multiplex Genome Editing Procedure.

As in a previous study, pHK463 expressing λ Beta protein was used to increase the single-strand oligonucleotide-mediated recombineering efficiency and *cas9*-integrated *E. coli* MG1655 was used for genome editing.[6] For genome editing, *E. coli* MG1655 was made into a competent cell after overexpression of λ Beta protein of pHK463 and Cas9 in the chromosome. At 30 °C, *E. coli* was cultured until OD_{600nm} = 0.4. Then, L-arabinose was added at a final concentration of 1 mM and incubated for 3 hours. The cultured cells were harvested, washed twice with 10% glycerol, resuspended, and stored at −80 °C.

Mutagenic oligonucleotides (100 or 500 pmol each) and sgRNA plasmids (200 ng) were electroporated into the cells under conditions of 25 *μ*F, 200 Ω, and 1.8 kV using a 0.1 cm electroporation cuvette (Bio-Rad). Thereafter, 950 mL of SOC medium was immediately added and recovered at 30 or 37 °C and 180 rpm. After 1 hour of recovery, cells were spread on LB or MacConkey agar (BD Difco, Cat. No. 281810, USA) plate containing chloramphenicol (12.5 *μ*g/mL) or spectinomycin (50 or 75 *μ*g/mL) and incubated at 30 or 37°C for 16 hours. MacConkey agar was used to check the fermentation type by adding D-galactose (0.5%, CAS No. 59-23-4), D-xylose (0.5%, CAS No. 58-86-6), or D-sorbitol (0.5%, CAS No. 50-70-4). Mutagenic oligonucleotides used for multiplex genome editing are listed in Table S4.

Calculation of Negative Selection and Editing Efficiency.

Surviving colonies (white + red colonies) were counted to determine whether target cleavage by the CRISPR-Cas9 system was successful. Editing efficiencies were calculated by counting the number of red and white colonies in MacConkey agar ([white colonies] / [white + red colonies]). After three repetitions of the experiment, two to four white colonies were randomly selected and confirmed by Sanger sequencing for each gene (*galK*, *xylB*, or *srlD*). For *cI* and *ilvG* editing, editing efficiency was calculated as the ratio of multiplex-edited colonies for both genes among 10 randomly selected colonies. Edited colonies were confirmed by Sanger sequencing of each two genes. Primers used for PCR amplification and Sanger sequencing are listed in Table S3.

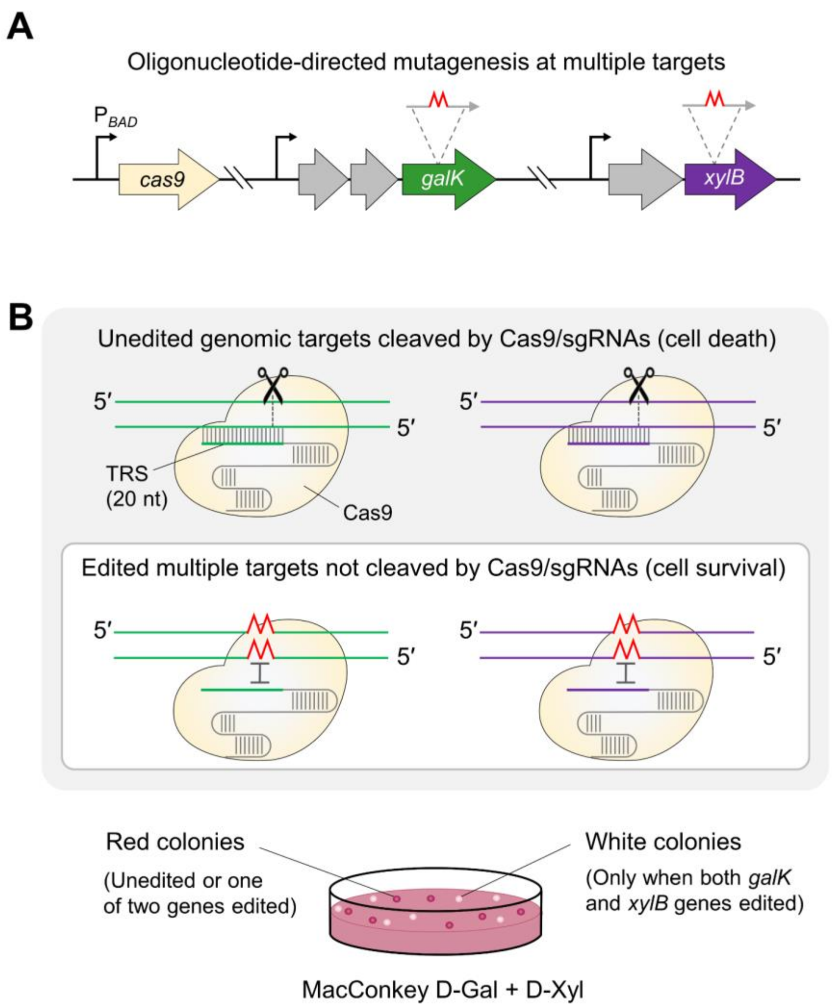

Supplementary Figure S1. Scheme of oligonucleotide-directed multiplex genome editing by CRISPR-Cas9 mediated negative selection. (**A**) Mutagenesis and negative selection process in multiple targets. Cas9 was expressed as L-arabinose inducible, and the mutagenic targets *galK* (green) and *xylB* (purple) are located in each operon in the genome. (**B**) Negative selection of multiplex-edited targets. The sgRNA/Cas9 complex cleaves the target where no mutation has been introduced. Edited targets are not cleaved by the sgRNA/Cas9 complex. Only cells in which mutations were introduced into both genes form white colonies in MacConkey agar containing D-galactose and D-xylose because the multiplex-edited cells lost the ability to metabolize both sugars.

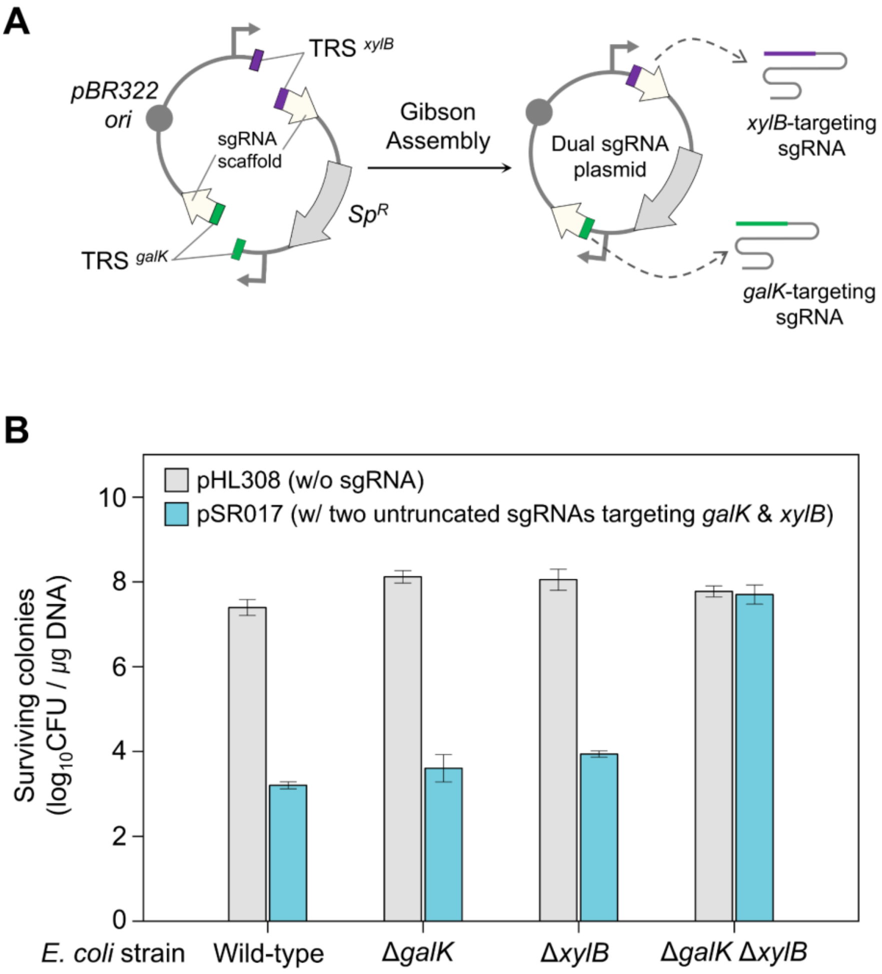

Supplementary Figure S2. Two-target multiplex genome editing using a sgRNA plasmid with two untruncated sgRNAs. (**A**) Construction of dual sgRNA plasmid targeting *galK* and *xylB*. Dual sgRNA plasmid was constructed by ligation of two DNA fragments harboring *ori* or spectinomycin resistance gene, with each end of the fragments identical to the corresponding N20 TRSs. Consequently, two different sgRNAs can be expressed from a single pSR017 plasmid. (**B**) *In vivo* cleavage efficiency of Cas9 with dual untruncated sgRNA plasmid. The number of surviving colonies with pHL308 indicates the transformation efficiencies of each strain, while the surviving colonies with pSR017 represent whether the genomic DNA cleavage occurred by sgRNA/Cas9 complexes in wild type, Δ*galK*, Δ*xylB*, and Δ*galK* Δ*xylB* *E. coli* strains. Each bar represents the mean value obtained from three independent experiments.

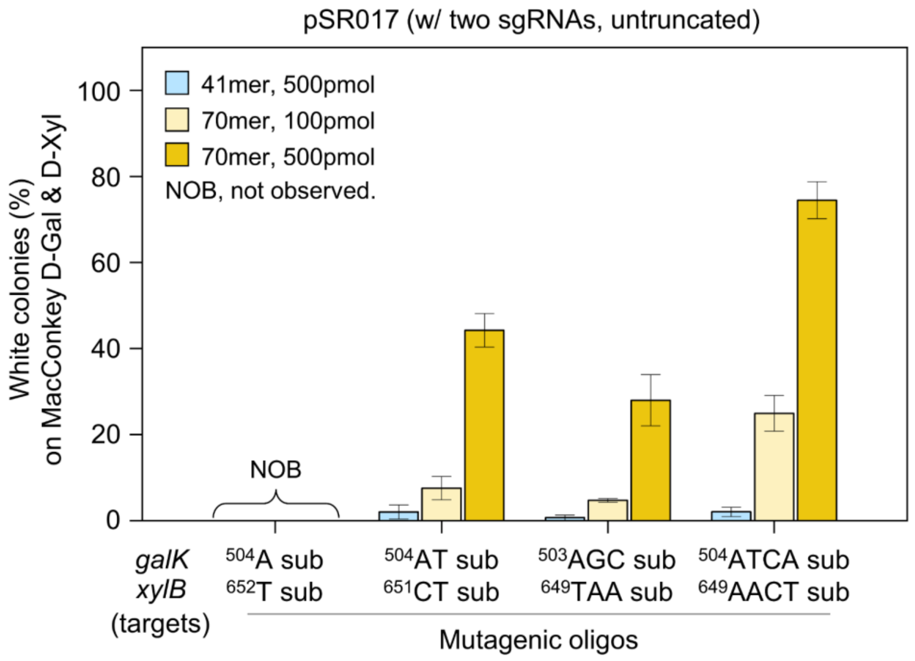

Supplementary Figure S3. Optimization of length and amount of oligonucleotides. Editing efficiencies of simultaneous 1–4 nt substitutions in *galK* and *xylB* were assessed in three different conditions of oligonucleotides. The oligonucleotide length was 41- or 70-mer, and the amount was 100 or 500 pmol. pSR017 expressing two untruncated sgRNAs targeting *galK* and *xylB* was co-transformed into Cas9-expressing *E. coli* with mutagenic oligonucleotides harboring one to four nucleotide(s) substitutions in *galK* or *xylB*. The percentage of white colonies to total colonies on MacConkey agar plate containing both D-galactose and D-xylose was calculated and defined as the editing efficiency.

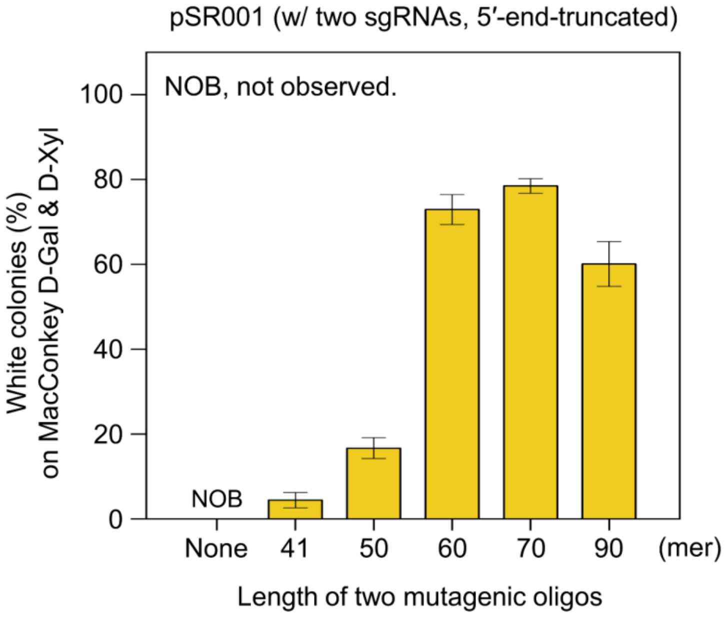

Supplementary Figure S4. Editing efficiency depending on the length of oligonucleotides. 100 pmol of mutagenic oligonucleotides with a length of 41- to 90-mer was transformed with a dual sgRNA plasmid pSR001. The two mutagenic oligonucleotides induce ^{504}T to A substitution in *galK* and ^{643}G to T substitution in *xylB*, respectively. Each bar represents the mean value obtained from three independent experiments.

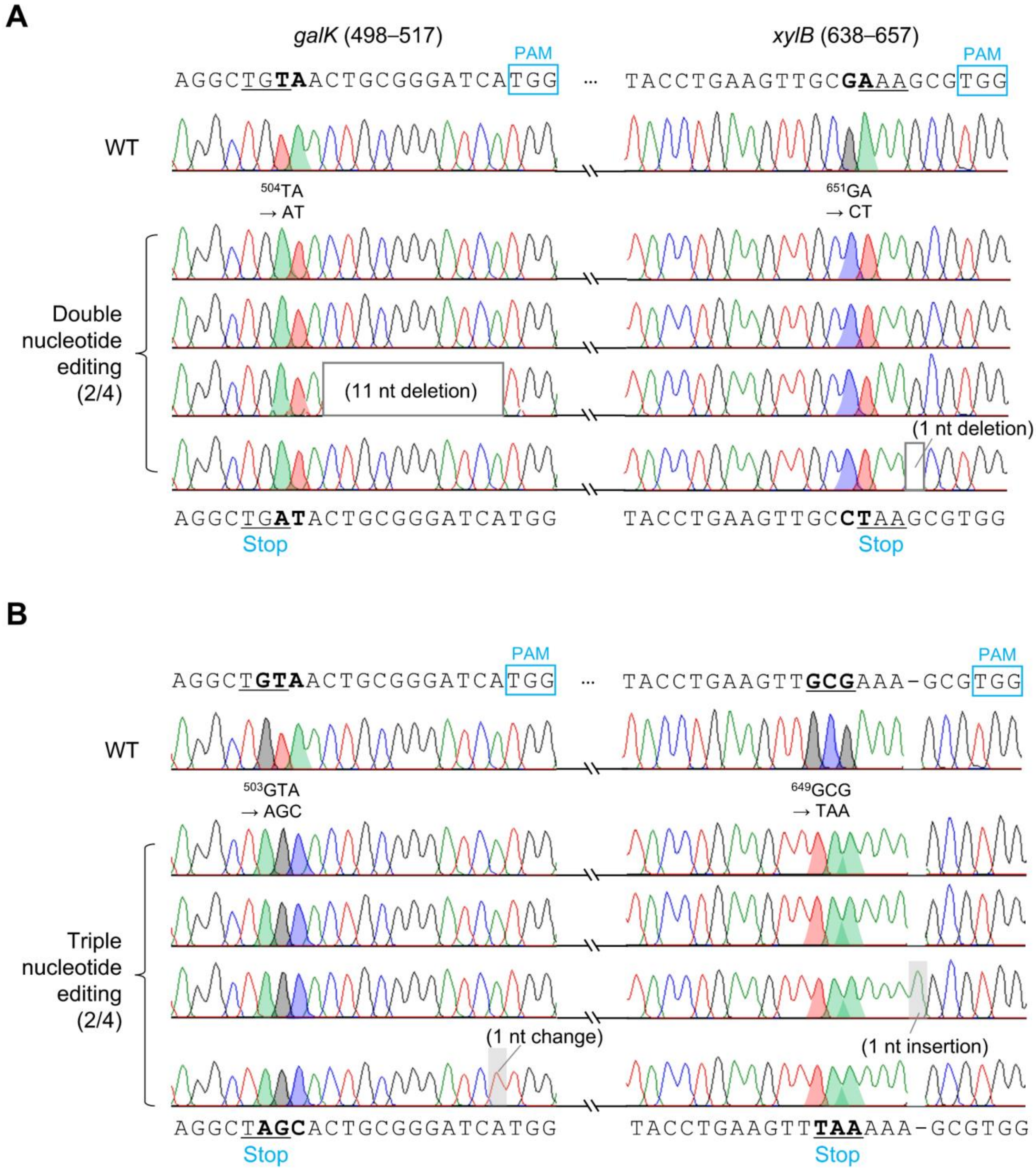

Supplementary Figure S5. Chromatograms of *galK* and *xylB* in double- and triple-nucleotide substituted cells obtained using two untruncated sgRNAs. (A) Double-nucleotide substitution, **(B)** Triple-nucleotide substitution. The underline indicates the position where a stop codon is generated by base substitution. Unwanted mutations were marked with a gray box.

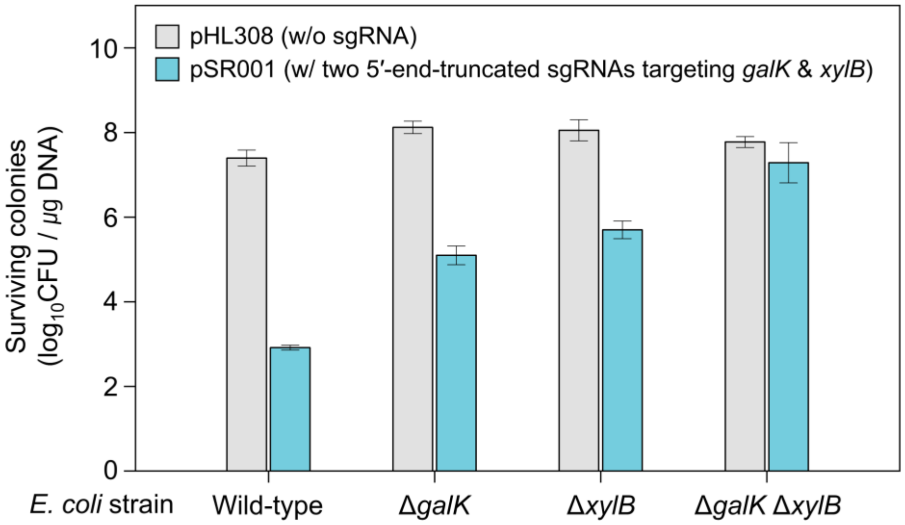

Supplementary Figure S6. Confirmation of surviving colonies by negative selection of dual 5′-end-truncated sgRNA plasmid targeting *galK* and *xylB*. The number of surviving colonies with pHL308 indicates the transformation efficiencies of each strain, while the surviving colonies with pSR001 represent whether the genomic DNA cleavage occurred by sgRNA/Cas9 complexes in wild type, Δ*galK*, Δ*xylB*, and Δ*galK* Δ*xylB* *E. coli* strains. Each bar represents the mean value obtained from three independent experiments.

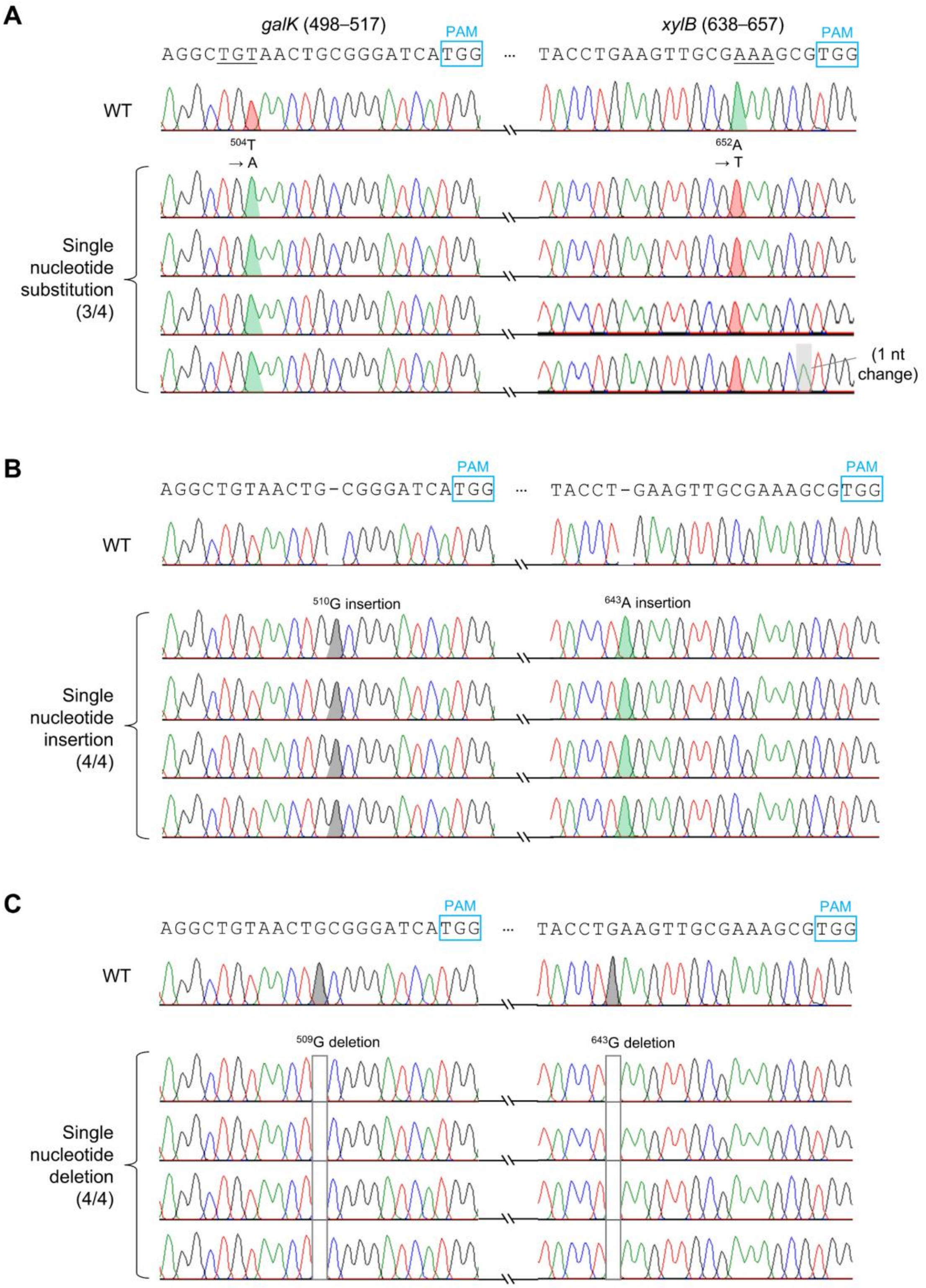

Supplementary Figure S7. Chromatograms of *galK* and *xylB* in single-nucleotide substituted-, inserted-, and deleted-cells obtained using truncated sgRNAs. (A) Single-nucleotide substitution, **(B)** Single-nucleotide insertion, and **(C)** Single-nucleotide deletion. Unwanted mutations were marked with a gray box.

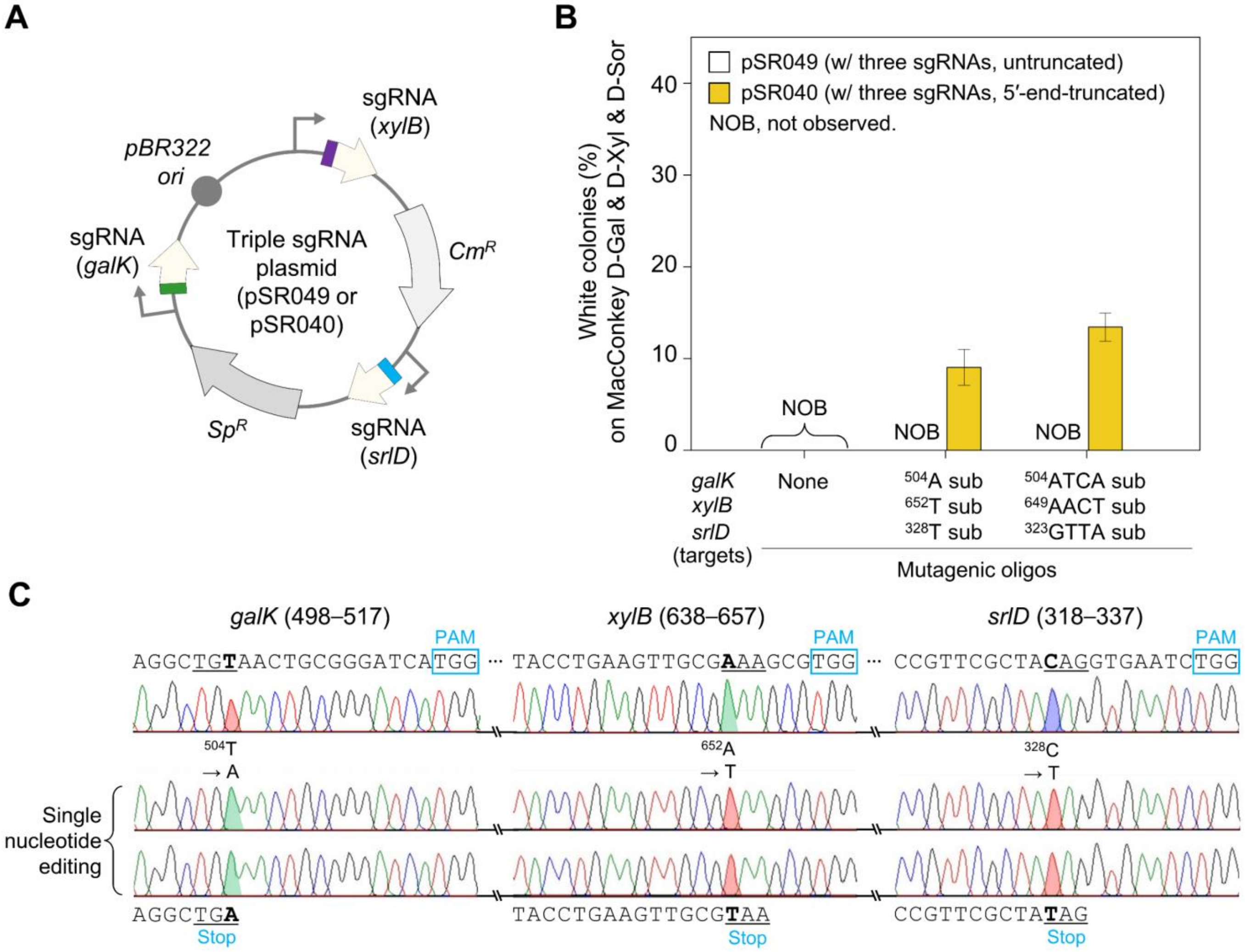

Supplementary Figure S8. Three-target single-nucleotide editing in *galK*, *xylB,* and *srlD* using 5′-end-truncated sgRNAs. (**A**) A plasmid carrying three sgRNAs targeting *galK*, *xylB*, and *srlD*. (**B**) Multiplex single- and quadruple-nucleotide substitutions using untruncated or 5′-end-truncated sgRNAs. (**C**) Sanger sequences of single-nucleotide edited *galK*, *xylB*, and *srlD* target sequences. The red-, green-, or blue-colored peaks with bolded letter indicate the target nucleotides for the multiplex genome editing.

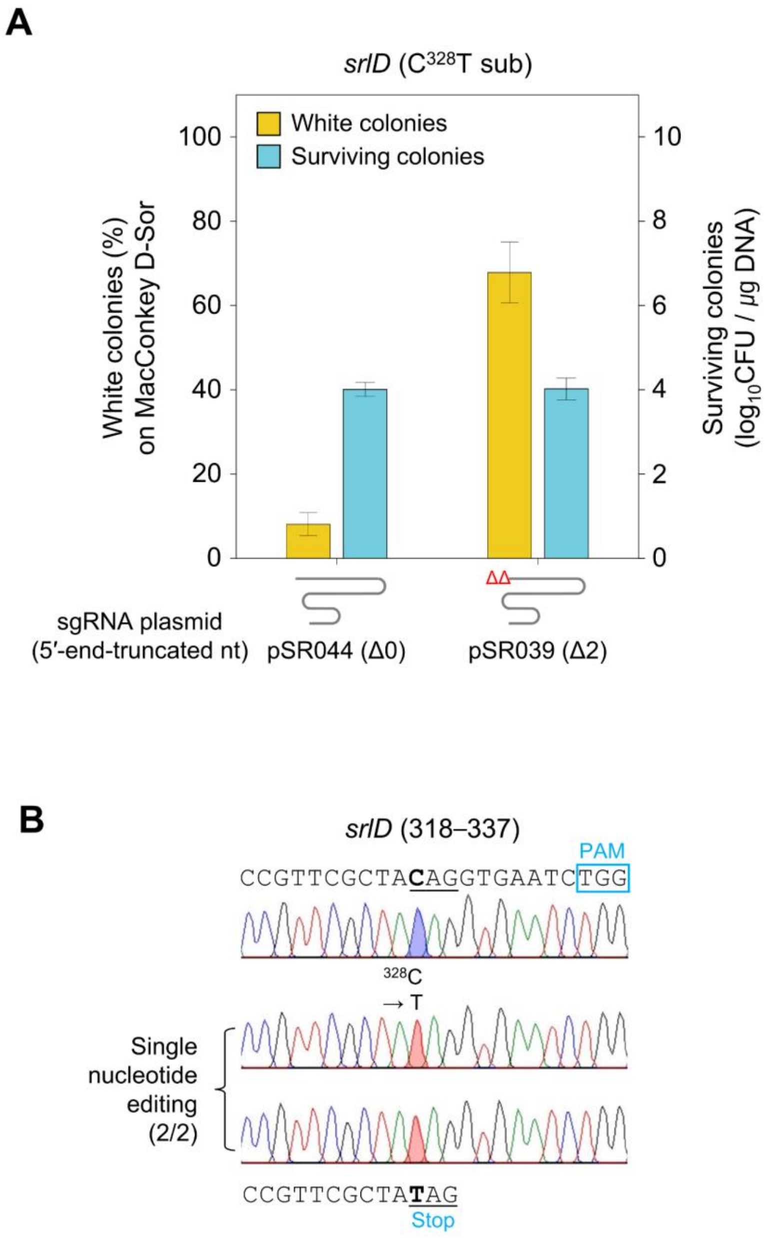

Supplementary Figure S9. Single-nucleotide editing in *srlD* target. (A) Comparison of single-nucleotide substitution efficiency at *srlD* target using untruncated or 5′-end-truncated sgRNAs. Each bar represents the mean value obtained from three independent experiments. **(B)** Chromatograms of single-nucleotide substituted *srlD* gene using truncated sgRNA.

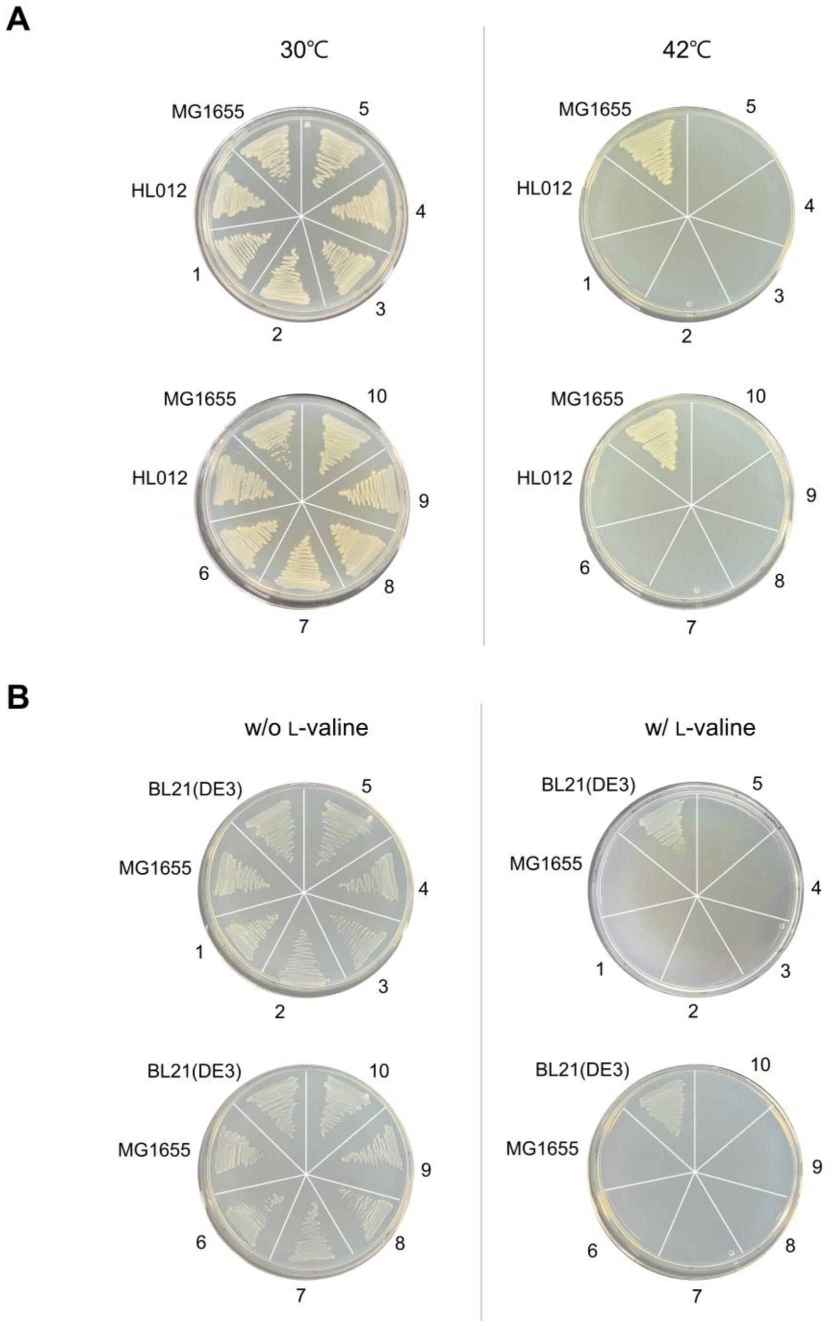

Supplementary Figure S10. Phenotypes of 10 randomly selected colonies after multiplex editing of *cI* and *ilvG* genes using untruncated sgRNAs. After multiplex editing by transforming dual untruncated sgRNA plasmid (pSR071) and mutagenic oligonucleotides, 10 colonies were randomly selected from LB agar. **(A)** MG1655 (λ^{-}), HL012 (λ cI^{857}), and 10 randomly selected colonies were grown on LB agar at 30 ℃ and 42 ℃. Unedited cI^{857} cells cannot grow at 42 ℃. **(B)** BL21(DE3) ($ilvG^{WT}$), MG1655 ($ilvG^{-}$), and the same 10 randomly selected colonies were grown on M9 D-glucose (0.4%) medium. L-valine (0.1 mM) was added if needed. Unedited $ilvG^{-}$ cells cannot grow on M9 agar containing D-glucose and L-valine due to L-valine toxicity.

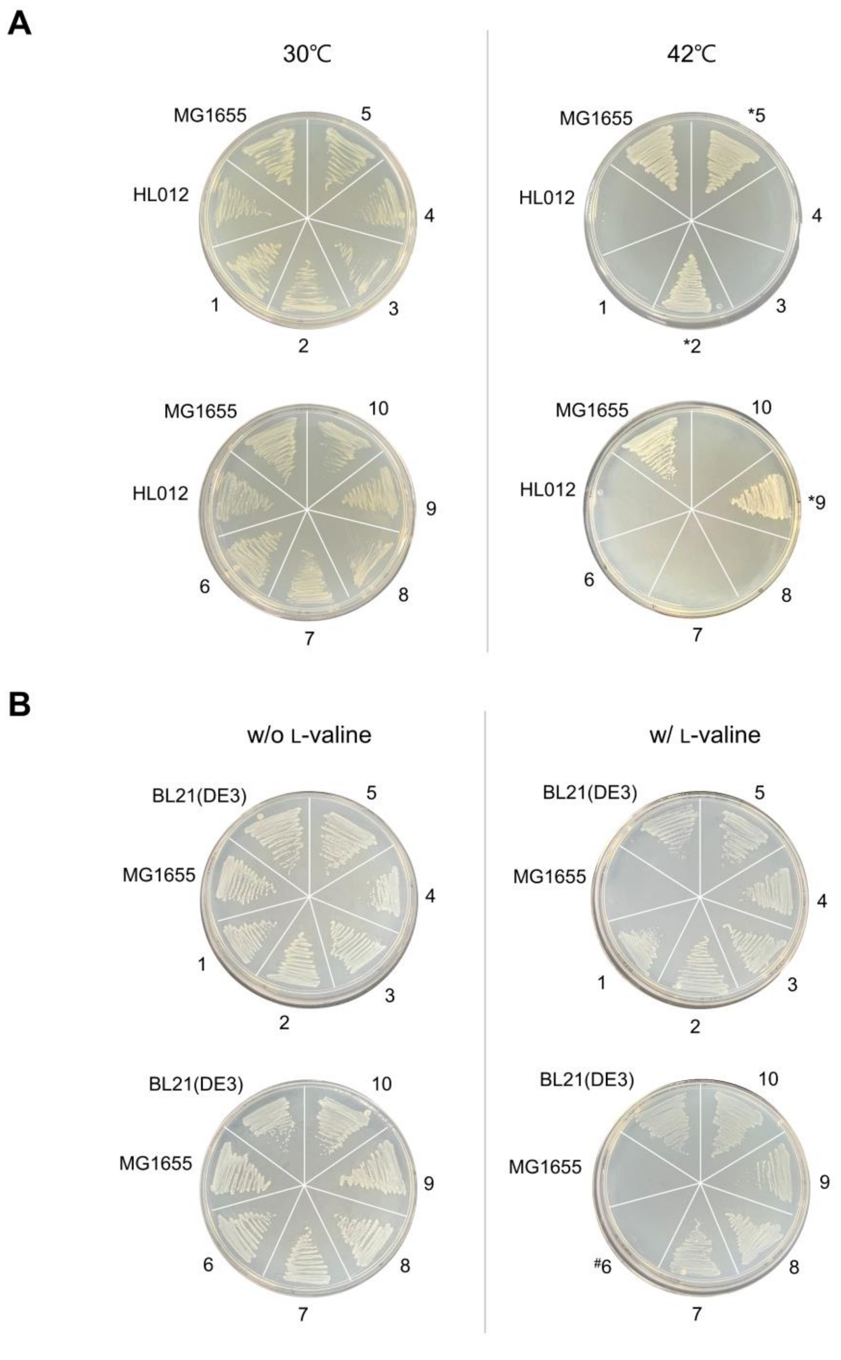

Supplementary Figure S11. Phenotypes of 10 randomly selected colonies after multiplex editing of *cI* and *ilvG* genes using 5′-end-truncated sgRNAs. After multiplex editing by transforming dual 5′-end-truncated sgRNA plasmid (pSR054) and mutagenic oligonucleotides, 10 colonies were randomly selected from LB agar. **(A)** MG1655 (λ⁻), HL012 (λ cI^{857}), and 10 randomly selected colonies were grown on LB agar. λ cI^{857} cells undergo lysis at 42 °C. *Edited λ cI^{WT} cells can grow at 42 °C. **(B)** BL21(DE3) ($ilvG^{WT}$), MG1655 ($ilvG^{-}$), and the same 10 randomly selected colonies were grown on M9 D-glucose (0.4%) medium. L-valine (0.1 mM) was added if needed. #Unedited $ilvG^{-}$ cells cannot grow on M9 agar containing D-glucose and L-valine due to L-valine toxicity.

Supplementary Table S1. Strains used in this study.

Name	Characteristics	Source/reference
Strain		
Escherichia coli		
BL21(DE3)	F^- *ompT gal dcm lon* $hsdS_B(r_B^- m_B^-)$ λ(DE3 *[lacI lacUV5-T7p07 ind1 sam7 nin5]*) [*malB*$^+$]$_{K\text{-}12}$(λ^S)	Laboratory stock
DH5α	*fhuA2 lac(del)U169 phoA glnV44 Φ80' lacZ(del)M15 gyrA96 recA1 relA1 endA1 thi-1 hsdR17*	Laboratory stock
MG1655	F^- *ilvG*$^-$ *rfb*-50 *rph*-1	Laboratory stock
HK1059	MG1655, *araBAD*::P_{BAD}-*cas9*-FRT-Km^R-FRT	(6)
JW0740	BW25113, *galK*::FRT-Km^R-FRT	(2)
JW2674	BW25113, *xylB*::FRT-Km^R-FRT	(2)
HL012	MG1655, *araBAD*::P_{BAD}-*cas9*-FRT-Km^R-FRT, λ *cI*^{857}	(1)
HL026	MG1655, *araBAD*::P_{BAD}-*cas9*-FRT	This study
SR004	MG1655, *araBAD*::P_{BAD}-*cas9*-FRT, *galK*::FRT-Km^R-FRT	This study
SR005	MG1655, *araBAD*::P_{BAD}-*cas9*-FRT, *xylB*::FRT-Km^R-FRT	This study
SR008	MG1655, *araBAD*::P_{BAD}-*cas9*-FRT, *xylB*::FRT	This study
SR009	MG1655, *araBAD*::P_{BAD}-*cas9*-FRT, *galK*::FRT-Km^R-FRT, *xylB*::FRT	This study
SR019	MG1655, *araBAD*::P_{BAD}-*cas9*-FRT-Km^R-FRT, *ilvG*^{WT} λ *cI*^{WT}	This study
Phage		
P1 *vir*	*vir* mutations	S. Adhya

Supplementary Table S2. Plasmids used in this study.

Plasmid	Characteristics	Source/reference
pACYC184	p15A *ori*, Cm^R, Tc^R	(4)
pCP20	pSC101 *ori*^{ts}, λ repressor, FLP gene, Amp^R, Cm^R	(5)
pHK463	pSC101 *ori*^{ts}, *araC*, λ *bet* gene, Amp^R	(6)
pTargetF	pBR322 *ori*, sgRNA target (^{1245}CATCGCCGCAGCGGTTTCAG^{1226} in *cadA*), Sp^R	Addgene #62226
pHL308	pBR322 *ori*, Sp^R	(7)
pHK459	pBR322 *ori*, sgRNA target (^{498}AGGCTGTAACTGCGGGATCA^{517} in *galK*), Sp^R	(8)
pHK470	pBR322 *ori*, sgRNA targets (^{498}AGGCTGTAACTGCGGGATCA^{517} in *galK,* ^{1245}CATCGCCGCAGCGGTTTCAG^{1226} in *cadA*), Sp^R	This study
pSR001	pBR322 *ori*, sgRNA targets (^{500}GCTGTAACTGCGGGATCA^{517} in *galK*, ^{640}CCTGAAGTTGCGAAAGCG^{657} in *xylB*), Sp^R	This study
pSR017	pBR322 *ori*, sgRNA targets (^{498}AGGCTGTAACTGCGGGATCA^{517} in *galK*, ^{638}TACCTGAAGTTGCGAAAGCG^{657} in *xylB*), Sp^R	This study
pSR020	pBR322 *ori*, sgRNA targets (^{500}GCTGTAACTGCGGGATCA^{517} in *galK*, ^{640}CCTGAAGTTGCGAAAGCG^{657} in *xylB*), Cm^R	This study
pSR039	pBR322 *ori*, sgRNA targets (^{320}GTTCGCTACAGGTGAATC^{337} in *srlD*), Sp^R	This study
pSR040	pBR322 *ori*, sgRNA targets (^{500}GCTGTAACTGCGGGATCA^{517} in *galK*, ^{318}CCGTTCGCTACAGGTGAATC^{337} in *srlD*, ^{640}CCTGAAGTTGCGAAAGCG^{657} in *xylB*), Cm^R, Sp^R	This study
pSR044	pBR322 *ori*, sgRNA targets (^{318}CCGTTCGCTACAGGTGAATC^{337} in *srlD*), Sp^R	This study
pSR049	pBR322 *ori*, sgRNA targets (^{498}AGGCTGTAACTGCGGGATCA^{517} in *galK*, ^{318}CCGTTCGCTACAGGTGAATC^{337} in *srlD*, ^{638}TACCTGAAGTTGCGAAAGCG^{657} in *xylB*), Cm^R, Sp^R	This study
pSR054	pBR322 *ori*, sgRNA target (^{193}GAATTTTTGTAAGCAATG^{210} in *cI*, ^{968}AGCCGTTAAATCAATGAC^{985} in *ilvG*), Sp^R	This study
pSR071	pBR322 *ori*, sgRNA target (^{191}GAGAATTTTTGTAAGCAATG^{210} in *cI*, ^{966}GCAGCCGTTAAATCAATGAC^{985} in *ilvG*), Sp^R	This study

Supplementary Table S3. Primers used in this study.

Name	Sequence (5′→3′)	Description
galT_half_F	GCAAATAGCTTCCTGCCTAACGAAGC	Confirmation of *galK*::Km^R cassette
xylA_F	CGTGGCGATGCGCAACTGGGCTGGGAC	Confirmation of *xylB*::Km^R cassette
KmR_ATG_out	ACCTGCGTGCAATCCATCTTGTTCAATCAT	Confirmation of Km^R cassette
Cas9_400up	AAGTTGAAGGGTAGTCCAGAAGATAACG	Confirmation of Km^R sequence removal by pCP20
ara_500dn	CCAGCCAGAAGGAGACTTCTGTCCCTTG	
galK_F	CATCAGCGTGACTACCATCCCTGCGTTG	PCR and sequencing primers for *galK* target
galK_R	CCAGCGAGACCTGACCGCAGAACAGGC	
xylB_F	GCTCAACGAGCAGGGTGAGGTGGTTGCTG	PCR and sequencing primers for *xylB* target
xylB_R	GATATCCGCCAGCATCTGACGCCAGTAC	
srlD_F	CTGGGTCATGTCACCCTGCGCTTCGA	PCR and sequencing primers for *srlD* target
srlD_R	GTAGTGGCGGAAGTCCTGTATGCCTG	
cI_F	AAACCATTCTTCATAATTCAATCCAT	PCR and sequencing primers for *cI* target
cI_R	TCTTTCAGGGTTATGCGTTGTTCCAT	
ilvG_F	GCATTCGACGTTGCCTGCTCAGGTCG	PCR and sequencing primers for *ilvG* target
ilvG_TGA137up_R	CCTGGTCTTTCCGGGTGATGTGTTGG	
pBR322ori_F	GGGAAACGCCTGGTATCTTTATAGTC	Sequencing primers for sgRNA cassette confirmation
Sm_ATG_out	GATACTGGGCCGGCAGGCGCTCCATTGCCC	Construction of sgRNA plasmids
Sm_TAA_out	GCAATGGAGCGCCTGCCGGCCCAGTATCAG	
gRNA_1F	TCGATTGGCTGAGCATATGCTGGATCCTTGACAGCTAGCTC	pHK470 plasmid construction
gRNA_2R	GATCCAGCATATGCTCAGCCAATCGACTGGCGAGCGGCATC	
gRNA_3F	ATCCCTACTCGAGCTCATAAGTTCCTATTCCGAAGTTCCGC	
gRNA_4R	AGGAACTTATGAGCTCGAGTAGGGATAACAGGGTAATAGAT	
CmR_pACYC184_F	TGATCGGCACGTAAGAGGTTCCAACTTTC	pSR020 plasmid construction
CmR_pACYC184_R	TTACGCCCCGCCCTGCCACTCATCGCAG	
pTarget_CmR_F	GAGTGGCAGGGCGGGGCGTAAGATGCCGCTCGCCAGTCGATTGGCTGAGC	
pTarget_CmR_R	GGAACCTCTTACGTGCCGATCAACTCGAGTAGGGATAACAGGGTAATAGATCTAAGC	

Supplementary Table S3. Primers used in this study (continued).

Name	Sequence (5′→3′)	Description
galK498,20_F	AGGCTGTAACTGCGGGATCAGTTTTAGAGCTAGAAATAGCAAG	pSR017, pSR049 plasmid construction
galK498,20_R	TGATCCCGCAGTTACAGCCTACTAGTATTATACCTAGGACT	
galK498,18_F	GCTGTAACTGCGGGATCAGTTTTAGAGCTAGAAATAGCAAGTT	pSR001, pSR040 plasmid construction
galK498,18_R	AAACTGATCCCGCAGTTACAGCACTAGTATTATACCTAGGAC	
xylB638,20_F	TACCTGAAGTTGCGAAAGCGGTTTTAGAGCTAGAAATAGCAAG	pSR017, pSR049 plasmid construction
xylB638,20_R	CGCTTTCGCAACTTCAGGTAACTAGTATTATACCTAGGACTG	
xylB638,18_F	CCTGAAGTTGCGAAAGCGGTTTTAGAGCTAGAAATAGCAAGTT	pSR001, pSR040 plasmid construction
xylB638,18_R	ACCGCTTTCGCAACTTCAGGACTAGTATTATACCTAGGACTG	
srlD318,20_F	CCGTTCGCTACAGGTGAATCGTTTTAGAGCTAGAAATAGC	pSR044, pSR049 plasmid construction
srlD318,20_R	GATTCACCTGTAGCGAACGGACTAGTATTATACCTAGGAC	
srlD318,18_F	GTGTTCGCTACAGGTGAATCGTTTTAGAGCTAGAAATAGC	pSR039, pSR040 plasmid construction
srlD318,18_R	ACGATTCACCTGTAGCGAACACTAGTATTATACCTAGGAC	
SpR_R	CGATACTTCGGCGATCACCGCTTCCCTC	pSR040, pSR049 plasmid construction
pBR322ori_TAA_out	TTCTGTGGATAACCGTATTACCGCCTTTGAGTGAGCTGATA	
pBR322_R	CACGCTGTAGGTATCTCAGTTCGGTG	
Lambda_cI191,18_F	GAATTTTTGTAAGCAATGGTTTTAGAGCTAGAAATAGCAAG	pSR054 plasmid construction
Lambda_cI191,18_R	CATTGCTTACAAAAATTCACTAGTATTATACCTAGGACTG	
ilvG966,18_F	AGCCGTTAAATCAATGACGTTTTAGAGCTAGAAATAGCAAG	
ilvG966,18_R	ACGTCATTGATTTAACGGCTACTAGTATTATACCTAGGACTG	
Lambda_cI191,20_F	GAGAATTTTTGTAAGCAATGGTTTTAGAGCTAGAAATAGCA	pSR071 plasmid construction
Lambda_cI191,20_R	CATTGCTTACAAAAATTCTCACTAGTATTATACCTAGGAC	
ilvG966,20_F	GCAGCCGTTAAATCAATGACGTTTTAGAGCTAGAAATAGCAAG	
ilvG966,20_R	GTCATTGATTTAACGGCTGCACTAGTATTATACCTAGGACTG	

Supplementary Table S4. Mutagenic oligonucleotides used in this study.

Name	Sequence (5′→3′)	Description
galKT504A	GAAAACCAGTTTGTAGGCTGAAACTGCGGGATCATGGATCA	Mutagenic oligonucleotides for *galK* ^{504}T to A substitution
galKT504A_50	AGCAGAAAACCAGTTTGTAGGCTGAAACTGCGGGATCATGGATCAGCTAA	
galKT504A_60	CAGGAAGCAGAAAACCAGTTTGTAGGCTGAAACTGCGGGATCATGGATCAGCTAATTTCC	
galKT504A_70	ACGGTCAGGAAGCAGAAAACCAGTTTGTAGGCTGAAACTGCGGGATCATGGATCAGCTAATTTCCGCGCT	
galKT504A_90	ATCGCGCTTAACGGTCAGGAAGCAGAAAACCAGTTTGTAGGCTGAAACTGCGGGATCATGGATCAGCTAATTTCC GCGCTCGGCAAGAAA	
galKTA504AT	GAAAACCAGTTTGTAGGCTGATACTGCGGGATCATGGATCA	Mutagenic oligonucleotides for *galK* ^{504}TA to AT substitution
galKTA504AT_70	ACGGTCAGGAAGCAGAAAACCAGTTTGTAGGCTGATACTGCGGGATCATGGATCAGCTAATTTCCGCGCT	
galKGTA503AGC	GAAAACCAGTTTGTAGGCTAGCACTGCGGGATCATGGATCA	Mutagenic oligonucleotides for *galK* ^{503}GTA to AGC substitution
galKGTA503AGC_70	ACGGTCAGGAAGCAGAAAACCAGTTTGTAGGCTAGCACTGCGGGATCATGGATCAGCTAATTTCCGCGCT	
galKTAAC504ATCA	GAAAACCAGTTTGTAGGCTGATCATGCGGGATCATGGATCA	Mutagenic oligonucleotides for *galK* ^{504}TAAC to ATCA substitution
galKTAAC504ATCA_70	CGGTCAGGAAGCAGAAAACCAGTTTGTAGGCTGATCATGCGGGATCATGGATCAGCTAATTTCCGCGCTC	
xylBG643T	TTACTGGTGCTTTGTTACCTTAAGTTGCGAAAGCGTGGGGT	Mutagenic oligonucleotides for *xylB* ^{643}G to T substitution
xylBG643T_50	CGAAATTACTGGTGCTTTGTTACCTTAAGTTGCGAAAGCGTGGGGTATGG	
xylBG643T_60	GGCAGCGAAATTACTGGTGCTTTGTTACCTTAAGTTGCGAAAGCGTGGGGTATGGCGACG	
xylBG643T_70	ACGAAGGCAGCGAAATTACTGGTGCTTTGTTACCTTAAGTTGCGAAAGCGTGGGGTATGGCGACGGTGCC	
xylBG643T_90	CCGCATTATACGAAGGCAGCGAAATTACTGGTGCTTTGTTACCTTAAGTTGCGAAAGCGTGGGGTATGGCGACGG TGCCAGTTGTCGCAG	
xylBA652T	CTTTGTTACCTGAAGTTGCGTAAGCGTGGGGTATGGCGACG	Mutagenic oligonucleotides for *xylB* ^{652}A to T substitution
xylBA652T_70	CGAAATTACTGGTGCTTTGTTACCTGAAGTTGCGTAAGCGTGGGGTATGGCGACGGTGCCAGTTGTCGCA	

- Target nucleotides for substitution and insertion are underlined.
- Deleted nucleotides are indicated in parenthesis.

Supplementary Table S4. Mutagenic oligonucleotides used in this study (continued).

Name	Sequence (5′→3′)	Description
xylBGA651CT<br>xylBGA651CT_70	CTTTGTTACCTGAAGTTGCCTAAGCGTGGGGTATGGCGACG<br>GCGAAATTACTGGTGCTTTGTTACCTGAAGTTGCCTAAGCGTGGGGTATGGCGACGGTGCCAGTTGTCGC	Mutagenic oligonucleotides for *xylB* ^{651}GA to CT substitution
xylBGCG649TAA<br>xylBGCG649TAA_70	TGCTTTGTTACCTGAAGTTTAAAAAGCGTGGGGTATGGCGA<br>AGCGAAATTACTGGTGCTTTGTTACCTGAAGTTTAAAAAGCGTGGGGTATGGCGACGGTGCCAGTTGTCG	Mutagenic oligonucleotides for *xylB* ^{649}GCG to TAA substitution
xylBGCGA649AACT<br>xylBGCGA649AACT_70	GCTTTGTTACCTGAAGTTAACTAAGCGTGGGGTATGGCGAC<br>AGCGAAATTACTGGTGCTTTGTTACCTGAAGTTAACTAAGCGTGGGGTATGGCGACGGTGCCAGTTGTCG	Mutagenic oligonucleotides for *xylB* ^{649}GCGA to AACT substitution
galK510Gins_70	CAGGAAGCAGAAAACCAGTTTGTAGGCTGTAACTGGCGGGATCATGGATCAGCTAATTTCCGCGCTCGGC	Mutagenic oligonucleotides for G insertion after *galK* ^{509}G
galK509Gdel_70	TCAGGAAGCAGAAAACCAGTTTGTAGGCTGTAACT(G)CGGGATCATGGATCAGCTAATTTCCGCGCTCGGCA	Mutagenic oligonucleotides for *galK* ^{509}G deletion
xylB643Ains_70	ACGAAGGCAGCGAAATTACTGGTGCTTTGTTACCTAGAAGTTGCGAAAGCGTGGGGTATGGCGACGGTGC	Mutagenic oligonucleotides for A insertion after *xylB* ^{642}T
xylB643Gdel_70	ACGAAGGCAGCGAAATTACTGGTGCTTTGTTACCT(G)AAGTTGCGAAAGCGTGGGGTATGGCGACGGTGCCA	Mutagenic oligonucleotides for *xylB* ^{643}G deletion
srlDC328T_70	CGACTTCCAGCTCGGCGATTTTGACCGTTCGCTATAGGTGAATCTGGTGGGTTATTTCCTGTGTGCGCGT	Mutagenic oligonucleotides for *srlD* ^{328}C to T substitution
srlDCGCT323GTTA_70	TCAGCGACTTCCAGCTCGGCGATTTTGACCGTTGTTAACAGGTGAATCTGGTGGGTTATTTCCTGTGTGC	Mutagenic oligonucleotides for *srlD* ^{323}CGCT to GTTA substitution
Lambda_cIA199G_70	CAATGCATTAAATGCTTATAACGCCGCATTGCTTGCAAAAATTCTCAAAGTTAGCGTTGAAGAATTTAGC	Mutagenic oligonucleotides for *cI* ^{199}A to G substitution
ilvG979ATins_70	ACGCAGCTGCGCGCAGTGTTGCTGCCAGTCATTGATATTTAACGGCTGCTGTAATGCTGGTAACAGAGCA	Mutagenic oligonucleotides for AT insertion after *ilvG* ^{978}T

- Target nucleotides for substitution and insertion are underlined.
- Deleted nucleotides are indicated in parenthesis.

REFERENCES

(1) Lee, H. J.; Kim, H. J.; Lee, S. J. Control of λ lysogenic *Escherichia coli* cells by synthetic λ phage carrying *cI*^{antisense}. *ACS Synth. Biol.* **2022**, *11*, 3829–3835.

(2) Baba, T.; Ara, T.; Hasegawa, M.; Takai, Y.; Okumura, Y.; Baba, M.; Datsenko, K. A.; Tomita, M.; Wanner, B. L.; Mori, H. Construction of *Escherichia coli* K-12 in-frame, single-gene knockout mutants: the Keio collection. *Mol. Sys. Biol.* **2006**, *2*, 2006.0008.

(3) Lee, H. J.; Kim, H. J.; Lee, S. J. Mismatch intolerance of 5´-truncated sgRNAs in CRISPR/Cas9 enables efficient microbial single-base genome editing. *Int. J. Mol. Sci.* **2021**, *22*, 6457.

(4) Chang, A. C.; Cohen, S. N. Construction and characterization of amplifiable multicopy DNA cloning vehicles derived from the P15A cryptic miniplasmid. *J. Bacteriol.* **1978**, *134*, 1141–1156.

(5) Cherepanov, P. P.; Wackernagel, W. Gene disruption in *Escherichia coli*: TcR and KmR cassettes with the option of Flp-catalyzed excision of the antibiotic-resistance determinant. *Gene* **1995**, *158*, 9–14.

(6) Lee, H. J.; Kim, H. J.; Lee, S. J. CRISPR-Cas9-mediated pinpoint microbial genome editing aided by target-mismatched sgRNAs. *Genome Res.* **2020**, *30*, 768–775.

(7) Lee, H. J.; Kim, H. J.; Lee, S. J. Miniature CRISPR-Cas12f1-mediated single-nucleotide microbial genome editing using 3´-truncated sgRNA. *CRISPR J.* **2023**, *6*, 52−61.

(8) Kim, B.; Kim, H. J.; Lee, S. J. Regulation of microbial metabolic rates using CRISPR interference with expanded PAM sequences. *Front. Microbiol.* **2020**, 282.
